# The effect of fertility stress on endometrial and subendometrial blood flow among infertile women

**DOI:** 10.1186/s12958-017-0236-7

**Published:** 2017-03-04

**Authors:** Yuezhi Dong, Yanna Cai, Yu Zhang, Yurong Xing, Yingpu Sun

**Affiliations:** Reproductive Medical Center, First Affiliated Hospital, Zhengzhou University, Zhengzhou, Henan China

**Keywords:** Infertility, Stress, 3D power Doppler ultrasound, Subendometrial flow index

## Abstract

**Background:**

To investigate the effect of fertility stress on endometrial and subendometrial blood flow among infertile women.

**Methods:**

This case–control study was conducted in The First Affiliated Hospital of Zhengzhou University. The fertility problem inventory (FPI) was adopted to evaluate fertility stress. Three-dimensional power Doppler ultrasonography (3D PD-US) was performed during the proliferative phase of the menstrual cycle (days 5–11) to measure endometrial thickness, pattern, endometrial and subendometrial volume (V), the vascularization index (VI), the flow index (FI) and the vascularization-FI (VFI) index. Then, 300 infertile women were separated into two groups (high-score group and low-score group) based on total FPI scores and 80 healthy women were selected as controls.

**Results:**

No differences were found among all three groups with regard to general characteristics, endometrial thickness, pattern, endometrial and subendometrial V, VI and VFI. The endometrial and subendometrial FIs associated with different stress levels significantly differed among the three groups (*F* = 33.95, *P <* 0.001; *F* = 44.79, *P <* 0.001, respectively). The endometrial and subendometrial FIs in the control group were significantly higher than those in the high-score group and low-score groups. The endometrial and subendometrial FIs in the low-score group were significantly higher than those in the high-score group. The total FPI score was closely related to the endometrial and subendometrial FIs (*r* = −0.304, *P* < 0.001; *r* = −0.407, *P* < 0.001, respectively).

**Conclusion:**

Fertility stress was associated with endometrial and subendometrial flow index. Whether fertility stress might affect pregnancy outcome by reducing endometrial and subendometrial blood flow requires further research.

## Background

Although in vitro fertilization (IVF) has helped many infertile couples become pregnant, those who choose to undergo IVF often suffer from the costs of medical treatment, the complexity of the procedures and unsuccessful cycles [1, 2]. Recently, researchers and clinicians have shown that psychological factors might reduce the chances of achieving pregnancy with IVF [3–5]. Previous studies have revealed a possible association between stress and reproductive outcomes. However, the specific pathways or mechanisms regarding how stress affects these outcomes have not been conclusively identified. Most studies have suggested that anxiety and depression negatively affect sex hormone, neuroendocrine, or immunologic functions related to pregnancy failure [5]. Other studies have suggested that fertility stress causes poorer responses to ovarian function or decreases the number of retrieved oocytes in IVF treatment [6, 7]. Moreover, Zhao showed that restraint-induced stress inhibits mouse implantation by impairing uterine receptivity and down regulating oestrogen (O) and progesterone (P) and heparin-binding epidermal growth factor (HB-EGF) [8]. Castelrn proposed that fertility stress affects uterine artery blood flow, consequently influencing IVF pregnancy outcomes [9]. However, few studies have used endometrial receptivity to address fertility stress when evaluating pregnancy outcomes. Moreover, how fertility stress affects pregnancy outcomes through the neuroendocrine and immunologic systems remains unclear. Furthermore, histologic and molecular studies are invasive [10, 11].

Endometrial receptivity allows blastocysts to implant into endometrial tissue and grow successfully. Three-dimensional power Doppler ultrasonography (3D PD-US) is a unique, non-invasive technique used to examine the vascularity of the entire endometrium or specific regions of interest [12]. Several sonographic parameters, such as endometrial thickness, pattern and volume (V), as well as the endometrial and subendometrial vascularization index (VI), flow index (FI) and vascularization-flow index (VFI), have long been considered markers of endometrial receptivity in clinical practice [13–16]. Furthermore, excellent endometrial and subendometrial blood supplies indicate endometrial receptivity and are related to successful IVF outcomes [17].

Therefore, we hypothesize that alterations in 3D PD-US imaging demonstrates the reduction in endometrial receptivity due to fertility stress. Specifically, we (i) tested whether the endometrial thickness, pattern and 3D PD-US parameters are possible indicators of the association between fertility stress and endometrial receptivity; (ii) aimed to identify which 3D PD-US parameters are related to fertility stress. The 3D PD-US parameters in this study refer to endometrial V and the endometrial and subendometrial VIs, FIs and VFIs.

## Methods

### Participants and eligibility

This cross-sectional study was conducted on infertility women who first came to the First Affiliated Hospital Reproductive Medicine Centerof Zhengzhou University in Henan province. A total of 300 infertility women who were diagnosed with pure tubal factor and unexplained factor from June 2015 to June 2016 were enrolled in this study.(hysterosalpingography (HSG) and hysteroscopy were used as the criterion for diagnosing of tubal factors.80 healthy women without fertility stress were selected as the control group at the Physical Examination Centerof the First Affiliated Hospitalof Zhengzhou University. All of the subjects (both infertility andhealthy women) follow the eligibility criteria: (i) aged 20 to 40 years old; (ii) self-reported menstrual cycle length of 21–39 days and in days 5–11 of their menstrual cycle; (iii) body mass index (BMI) less than 24.99 kg/m^2^; (iv) No smoking; (v) No thyroid dysfunction, hyperprolactinemia, adrenal hyperthyroidism and other endocrine diseases,no hypertension, diabetes;(vi) and able to understand Chinese well enough to complete the questionnaires. The exclusion criteria were (i) hydrosalpinx; (ii) no use of hormonal drugs within the past three months; (iii) a history of pelvic surgery; (iv) the experience of a major life event over the past 12 months or have mental disorder. The Clinical Ethics Committee, First Affiliated Hospital of Zhengzhou University, approved this study.

### Assessment of fertility stress

Perceived fertility stress was assessed using the fertility problem inventory (FPI) [18]. This measure shows satisfactory reliability and validity, with a Cronbach’s alpha coefficient ranging from 0.77-0.93. The Cronbach’s alpha coefficient in this study was 0.79. The inventory contains 46 items across 5 domains including social concerns, sexual concerns, relationship concerns, need for parenthood and rejection of a childfree lifestyle. Each item was scored on a 6-point Likert scale in which 1 denotes “I disagree” and 6 denotes “I totally agree”. A greater total score indicated a higher level of perceived psychological stress. This inventory has been widely used as a screening tool in clinical practice [19]. The Mandarin version of the FPI (M-FPI) has been used effectively to evaluate infertile couples in China [20].

### Transvaginal 3DPD-US examination

Given the variability associated with ovulation, all of the included women participated on menstrual days 5–11 to avoid additional selection variation. The patients and vaginal probe were kept as still as possible during the Volume acquisition. A single investigator performed all the examinations with a 5- to 9-MHz endocavitary transducer using a Voluson E8 3D PD-US acquisition device (GE Healthcare, Kretz, Zipf, Austria) to avoid interoperator bias. First, endometrial thickness and endometrial echogenicity were visualized in 2D B-mode. The maximum thickness of the endometrium was measured via a longitudinal section at the maximum anteroposterior dimension. Then, the 3D PD-US mode was activated to ensure that the entire uterine Volume obtained included the entire subendometrium. The settings were as follows: sweep angle, 120°; quality, low; wall motion filter, low 1; pulse repetition frequency, 0.9 kHz; and rotating angles, 15°. As a result, 12 contour planes were obtained for each endometrium. Once the Volume measurement was complete, the manual mode of the virtual organ computer-aided analysis (VOCAL) software for the 3D PD-US histogram instrument was used to calculate the Volume and the VI, FI and VFI within the endometrium. The endometrial Volume was drawn by hand along the endometrial outline. The area of interest within the subendometrium was the region 10 mm from the endometrial border, and it was obtained via editing in shell imaging [21]. The subendometrial Volume, VI, FI and VFI were likewise calculated. The vascularization index, which representedthe density of vessels in the tissue and wasexpressed as a percentage. The flow index was thought to express the average intensity of flow. The vascularization-flow index was a combination ofvascularity and flow intensity.

300 infertile females were equally separated into two groups according to their total FPI scores: patients in the high-score group (*n* = 150) had higher total FPI scores, which means have greater fertility stress.whereas patients in the low-score group (*n* = 150) had lower total FPI scores,which means have lower fertility stress. A total of 80 healthy females served as controls. The sample size was calculated using PASS11.0 software(PASS 11 citation: Hintze J (2011). PASS 11. NCSS, LLC. Kaysville, Utah, USA). In this study,The power is 100% and the *p*-value, is set to be less than 0.05.

### Statistical analyses

All the data were analyzed using SPSS 17.0 (SPSS Inc., Chicago, IL, USA). A single factor variance analysis was used to compare the differences of age, infertility duration, examination date and 3D-power Doppler characteristics; Means and SDs were computed for all FPI scores. U Mann–Whitney test were performed to compare the different proportions of residence, types of infertility, infertility causes and childbearing history across the different levels of psychological stress. Compare 3 independent samples were run by single factor variance. Pearson correlation coefficient (*r*) was used to determine the relationship between the FPI scores and the 3D PD-US characteristics. The significance level for all analyses was *P* < 0.05.

## Results

General information summarized in Table 1. The U Mann–Whitney testresults showed there were no differences among the 3 groups in terms of age, residence, infertility duration, infertility type, cause of infertility, childbearing history, examination date or menstrual cycle (*P* > 0.05).

**Table 1:** General characteristics for the three groups

Parameter	Control (*n* = 80)	Low-scoregroup (*n* = 150)	High-scoregroup (n = 150)
Age (y)	31.5 ± 4.6	29.6 ± 4.1	30.1 ± 4.5
Residence			
Urban	41 (51.2%)	66 (44.0%)	70 (46.6%)
Rural	39 (48.7%)	84 (56.0%)	80 (53.4%)
Infertility duration (y)	—	1.91 ± 0.80	2.05 ± 0.94
Type of infertility			
Primary infertility	—	95 (63.3%)	92 (61.3%)
Secondary infertility	—	55 (36.6%)	58 (38.6%)
Cause of infertility			
Female	—	75 (50.0%)	84 (56%)
Male	—	34 (22.6%)	35 (23.3%)
Mixed	—	21 (14.0%)	16 (10.6%)
Unexplained	—	20 (13.3%)	15 (10.0%)
History of childbearing			
Yes	14 (17.5%)	24 (16.0%)	20 (13.3%)
No	66 (82.5%)	126 (84.0%)	130 (86.7%)
Examination date, menstrual cycle (d)	9.60 ± 2.25	9.68 ± 2.22	9.61 ± 2.20

Table 2 shows the FPI scores of infertility women. The total FPI scores of the high-score group were 186.60 ± 17.931,ranging from 165 to 242. The total FPI scores of the low-score group were 143.44 ± 16.617,ranging from 97 to 164.

**Table 2:** FPI scores of the infertility women

FPI scores	High-score group (*n* = 150)	Low-score group (*n* = 150)
Social concern	37.26 ± 6.452	26.61 ± 6.388
Relationship concern	36.55 ± 7.449	26.46 ± 6.992
Need for parenthood	50.53 ± 6.366	42.50 ± 8.611
Rejection of childfree lifestyle	34.81 ± 7.361	29.85 ± 6.859
Sexual concern	27.86 ± 5.616	18.02 ± 5.710
Total	186.60 ± 17.931	143.44 ± 16.617

We also compared the clinical characteristics, including the endometrial thickness, volume, endometrial and subendometrial vascularization indexes, flow indexes, and vascularization-flow indexes. The scores of the three groups were similar in terms of endometrial thickness, endometrial and subendometrial Volume and vascularization index and vascularization-flow indexes (*P* > 0.05); however, the endometrial and subendome trial flow indexes significantly differed (*P* < 0.001). The endometrial and subendometrial flow indexes were significantly higher in the control group than in the high-score group and low-score groups. The endometrial and subendometrial flow indexes of the low-score group were significantly higher than those of the high-score group (Table 3).

**Table 3:** The 3D PD-US characteristics of the three groups

Parameter	Control (*n* = 80)	Low-scoregroup (*n* = 150)	High-score group (*n* = 150)
Endometrial thickness (mm)	7.38 ± 2.07	7.42 ± 2.58	6.93 ± 2.31
Endometrial volume (cm^3^)	2.36 ± 0.97	2.16 ± 1.08	2.33 ± 0.97
Endometrial vascularization index (%)	2.92 ± 3.30	3.43 ± 3.93	3.49 ± 3.44
Endometrial flow index	24.61 ± 6.10**	20.59 ± 4.98**	17.98 ± 6.27**
Endometrial vascularization-flow index	0.75 ± 0.96	0.63 ± 0.74	0.69 ± 0.73
Subendometrial volume	33.17 ± 7.54	30.52 ± 10.40	31.30 ± 8.10
Subendometrial vascularization index (%)	9.93 ± 8.10	10.03 ± 6.37	9.64 ± 5.01
Subendometrial flow index	31.83 ± 4.82**	27.83 ± 4.89**	24.86 ± 5.89**
Subendometrial vascularization-flow index	2.53 ± 1.88	2.62 ± 1.59	2.70 ± 1.35

**: *P* < 0.001

Single factor variance was used to compare 3 groups of endometrial and subendometrial blood flow indexs

Table 4 shows that the correlation coefficients between the need for parenthood,total FPI score and endometrial flow indexwere significantly different (*P* < 0.001). There were significant correlations between social concern, relationship concern, the need for parenthood, sexual concern, total FPI score and subendometrial flow index among infertile women (*P* < 0.001). However, the total FPI score, endometrial thickness and endometrial echogenicity, as well as the endometrial and subendometrial vascularization indexes and vascularization-flow indexes, were not significantly correlated (*P* > 0.05).

**Table 4:** The relationship between FPI scores and the 3D PD-US characteristics (*r*)

	Social concern	Relationship concern	Need for parenthood	Rejection of childfree lifestyle	Sexual concern	Total FPI score
Endometrial thickness	0.048	−0.044	−0.027	−0.02	0.06	0.006
Endometrial echogenicity	−0.029	−0.018	0.034	−0.052	−0.11	−0.043
Endometrial V	0.105	0.009	0.081	0.025	0.020	0.072
Endometrial VI	0.007	0.049	0.092	−0.021	−0.035	0.031
Endometrial FI	−0.233**	−0.220**	−0.237**	−0.153**	−0.183**	−0.304**
Endometrial VFI	0.016	0.077	0.088	−0.028	0.012	0.052
Subendometrial V	−0.017	−0.023	0.107	0.078	−0.047	0.029
Subendometrial VI	−0.035	0.013	0.107	−0.100	−0.111	−0.031
Subendometrial FI	−0.350**	−0.313**	−0.229**	−0.195**	−0.291**	−0.407**
Subendometrial VFI	−0.010	0.019	0.111	−0.110	−0.087	−0.016

*V*, Volume; *VI*, Vascularization index; *FI*, Flow index; *VFI*, Vascularization-flow index

**: *P* < 0.001

Pearson correlation coefficient (r) was used to determine the relationship between the FPI scores and the 3D PD-US characteristics

## Discussion

Our study explored the effect of fertility stress on endometrial and subendometrial blood flow, providing a comprehensive assessment that included endometrial thickness, endometrial pattern and Volume as well as endometrial and subendometrial VIs, FIs and VFIs. To the best of our knowledge, this study is the first to identify that the endometrial and subendometrial FIs significantly differed among these three groups and that fertility stress was correlated with endometrial and subendometrial FIs.

One study showed that neither age nor type of infertility affected endometrial or subendometrial blood flow [22]. However, other studies have shown that the FI is significantly lower among women older than 31 but significantly higher in mothers. The VI and VFI increased during the proliferative period and peaked three days after ovulation [23]. We compared the general characteristics among the 3 groups and did not find any significant differences. In addition, we compared the endometrial and subendometrial blood FIs, which were shown to significantly differ among the three groups. More importantly, we found that fertility stress was negative correlated with the endometrial and subendometrial FIs. Kupesic suggested that patients with greater endometrial FIs were associated with higher pregnancy rates [24]. This supposition is consistent with a study by Schild who also showed that subendometrial FI was the most important factor for predicting pregnancy outcomes [25]. Moreover, the correlation coefficient regarding the relationship between fertility stress and the endometrial FIs was less than that between stress and the subendometrial FI; this finding might be because the blood flow of the myometrium within the subendometrium directly affects the blood supply of the endometrium.

However, the mechanisms through which fertility stress affects the endometrial and subendometrial FIs remain unclear. we submit the following hypotheses. First, the activation of the stress response causes a series of neuroendocrine system reactions that are regulated by the two main neuroendocrine axes: the hypothalamic-pituitary-adrenocortical (HPA) axis and the sympathetic adrenomedullary (SAM) system [26]. They are associated with increased concentrations of glucocorticoids and catecholamines [27]. Stress can excite catecholamines alpha receptors and cause vasoconstriction, thereby reducing uterusblood flow [9]. Second,Corticotropic-releasing hormone (CRH) activates the HPA axis when stress is present in the body. Stress-induced HPA axis hyperfunction, causing hypothalamic-pituitary-ovarianaxisaxis (HPO) dysfunction or disorder of which the most important effect is to reduce the Gonadotropin - releasing hormone (GnRH) pulse secretion and then reduce the luteotropic hormone (LH) secretion. Moreover, CRH can not only reduce the basal level of estrogen but also inhibit Follicle stimulating hormone(FSH) synthesis of O. When the levels of oestrogen drop, the uterine basal and spiral arteries contract, followed by a rise in peripheral vascular resistance. Blood flow distribution is then reduced [28]. Keratinocyte growth factor (KGF-2), which originates from uterine stromal cells, is upregulated by the action of P. Slayden suggest that KGF-2 may affect embryo implantation and pregnancy outcome by increasing the blood supply to the myometrium and endometrium [29]. When the levels of P and KGF-2 drop, the blood supply to the endometrium and muscle layer decreases. Finally, stress can also cause persistent endothelial cell dysfunction,increase endometrial vascular permeability, reduced prostaglandin E2 (PG E2) release, leading to vasodilation decreased and contraction increased. Moreover, Carolpointed out that angiotensin II can promote endometrial vasoconstriction, resulting in blood volume decreased [30]. Bernatovasuggested that stress affects the neurotransmitter metabolism of Nitric oxide (NO), acetylcholine and serotonin, which eventually leads to decreases in the endometrial blood flow [31].

The present study has several strengths. First, our results provide new insights into the impact of fertility stress on pregnancy outcomes. Moreover, rather than creating an animal model, we used 3D PD-US and found that fertility stress was associated with endometrial and subendometrial FIs in women. Furthermore, we proposed a possible mechanism regarding how fertility stress affects the endometrial and subendometrial FIs, which provided a the oretical evidence for the impact of fertility stress on endometrial and subendometrial blood flow.

This study has several limitations. First, The limited number of FPI items made it extremely difficult to determine whether other factors affect fertility stress. Therefore, future studies that explore the role that stress plays on endometrial and subendometrial FIs should use a collection of stress biomarkers [32],and questionnaire data. Another weakness was our observational design. We only found that fertility stress is closely related to endometrial and subendometrial FIs. Whether stress affects pregnancy outcomes requires future longitudinal research. Finally, This study is only speculated and that the impact of fertility stress on endometrial and subendometrial blood flow lack direct evidence.

## Conclusions

This study demonstrates that fertility stress is closely associated with endometrial and subendometrial FIs, but it is not related to endometrial thickness, type or endometrial and subendometrial VIs and VFIs. Fertility stress might reduce the endometrial receptivity by lowering the endometrial and subendometrial FIs. Therefore, we should pay more attention to women's fertility stress andto taking effective psychological interventions to reduce thefertility stress may help improve the pregnancy outcomes.

## Abbreviation

3D PD-US: Three-dimensional power Doppler ultrasonography
BMI: Body mass index
CRH: Corticotropic-releasing hormone
FI: Fow index
FPI: The fertility problem inventory
FSH: Follicle stimulating hormone
GnRH: Gonadotropin - releasing hormone
HB-EGF: Heparin-binding epidermal growth factor
HPA: The hypothalamic-pituitary-adrenocortical
HPO: Hypothalamic- pituitary-ovarianaxis
IVF: In vitro fertilization
KGF-2: Keratinocyte growth factor
LH: Luteotropic hormone
M-FPI: The Mandarin version of the FPI
N: Nitric oxide.
O: Oestrogen
P: Progesterone
PG E2: Prostaglandin E2
SAM: The sympathetic adrenomedullary
V: Volume
VI: Vascularization index
VFI: Vasculariza tion-flow index
VOCAL: The virtual organ computer-aided analysis
